# Environmental Quality, Renewable Energy, and Life Expectancy in Gulf Cooperation Council Countries

**DOI:** 10.3390/ijerph23060750

**Published:** 2026-06-03

**Authors:** Ihsen Abid

**Affiliations:** Department of Finance, College of Business, Imam Mohammad Ibn Saud Islamic University (IMSIU), Riyadh 11564, Saudi Arabia; isbaklouti@imamu.edu.sa

**Keywords:** life expectancy, environmental quality, renewable energy, economic growth, government health expenditure, CO_2_ emissions

## Abstract

**Highlights:**

**Public health relevance—How does this work relate to a public health issue?**

- Environmental degradation and fossil fuel dependence may negatively affect population health and longevity in Gulf Cooperation Council (GCC) countries.
- Renewable energy transition and healthcare investment are increasingly important for sustainable public health outcomes in these countries.

**Public health significance—Why is this work of significance to public health?**

- The study provides dynamic panel evidence on how environmental, economic, and healthcare factors jointly influence life expectancy in the region.
- The findings highlight the adverse role of CO_2_ emissions and the positive contribution of renewable energy consumption and government health expenditure to longevity.

**Public health implications—What are the key implications or messages for practitioners, policy makers, and/or researchers in public health?**

- Policymakers should strengthen renewable energy strategies and environmental regulations to improve long-term population health outcomes.
- Expanding preventive healthcare investment and improving equitable healthcare access are essential for increasing life expectancy in the GCC countries.

**Abstract:**

Life expectancy is a key indicator of public health and sustainable development in Gulf Cooperation Council (GCC) countries, where rapid economic growth, urbanization, and fossil-fuel dependence create environmental and health challenges. This study examines the determinants of life expectancy in six Gulf Cooperation Council countries from 2000 to 2023, focusing on death rates, renewable energy consumption, gross domestic product (GDP) per capita growth, government health expenditure, and carbon dioxide (CO_2_) emissions. The empirical strategy combines cross-sectional dependence and slope heterogeneity tests, second-generation panel unit root tests, panel cointegration analysis, and a dynamic System Generalized Method of Moments (System GMM) estimator, with Driscoll–Kraay fixed-effects estimates used for robustness. The results show that higher death rates significantly reduce life expectancy, whereas renewable energy consumption and government health expenditure improve longevity. GDP per capita growth has a modest positive effect, while CO_2_ emissions negatively affect life expectancy, confirming the adverse public health consequences of environmental degradation. Robustness checks support the reliability of the main findings. Overall, the evidence highlights the need for integrated policies that combine clean energy transition, stronger environmental regulation, preventive healthcare investment, and sustainable urban development to improve long-term health outcomes in resource-dependent economies in the region.

## 1. Introduction

Life expectancy is a fundamental indicator of public health and socioeconomic development, reflecting the combined influence of healthcare systems, environmental conditions, and economic structures. Over recent decades, global gains in life expectancy have been largely driven by medical advancements, rising incomes, and improvements in living standards [1,2]. Nevertheless, pronounced disparities persist across regions, particularly in economies undergoing rapid structural and environmental transitions. For high-income countries seeking to sustain long-term development, coordinated progress across economic, environmental, and social domains is essential for improving population health and overall well-being [3]. In recent years, environmental health concerns have become increasingly central to public health debates, particularly due to the growing burden of air pollution, climate change, and fossil fuel dependence on human health outcomes. Environmental degradation is now recognized as a major contributor to respiratory diseases, cardiovascular disorders, premature mortality, and declining quality of life, especially in rapidly urbanizing economies.

The Gulf Cooperation Council region—comprising Bahrain, Kuwait, Oman, Qatar, Saudi Arabia, and the United Arab Emirates—has experienced profound economic and demographic transformations driven by hydrocarbon wealth, rapid urbanization, and technological advancement. While these countries benefit from high income levels and relatively advanced healthcare infrastructure, they also face mounting health and environmental challenges. These include the rising prevalence of non-communicable diseases (NCDs), elevated mortality from road traffic accidents, and environmental degradation associated with fossil fuel dependence and high per capita CO_2_ emissions [4]. Such factors jointly shape life expectancy dynamics and raise concerns about the long-term sustainability of health outcomes in the region. Moreover, GCC countries are among the world’s highest per capita carbon emitters, reflecting the heavy reliance on fossil-fuel-based energy systems and energy-intensive urban development models. These environmental pressures have intensified concerns regarding environmental health, sustainable urban living, and long-term healthcare burdens.

Environmental quality has emerged as a critical determinant of population health. Extensive evidence links air pollution and carbon-intensive energy systems to respiratory and cardiovascular diseases, premature mortality, and reduced life expectancy [5]. Conversely, transitions toward renewable energy sources can improve air quality and generate significant health co-benefits [6]. In parallel, economic growth and public health expenditure influence longevity through improved healthcare access, infrastructure, and living conditions, although their effectiveness depends on how resources are allocated and integrated with environmental policies. Renewable energy transition is therefore increasingly viewed not only as an environmental objective but also as a public health strategy capable of reducing pollution-related diseases and improving population well-being.

Despite this growing body of literature, empirical evidence that jointly integrates environmental quality, energy transition, and health outcomes within a unified dynamic framework remains limited, particularly in the context of high-income, resource-dependent economies such as the GCC countries. Most existing studies either focus on environmental–economic linkages or health expenditure effects in isolation, without capturing their combined and potentially interdependent impact on life expectancy. In addition, many previous studies rely on static econometric frameworks that fail to account for endogeneity, cross-sectional dependence, slope heterogeneity, and dynamic persistence, potentially leading to biased and inconsistent results.

This study investigates the determinants of life expectancy in the GCC countries over the period 2000–2023, focusing on five key factors: death rate (DR), renewable energy consumption (REN), GDP per capita growth (GDP), government health expenditure (GHE), and CO_2_ emissions (CO_2_). In doing so, it moves beyond conventional single-dimension analyses by explicitly modeling the joint economic–environmental–health nexus within a dynamic panel-data framework. While global studies document inverse relationships between mortality rates and life expectancy [2,7] and adverse health effects of environmental pollution [5], evidence for the region remains fragmented and lacks a comprehensive empirical treatment.

The novelty of this study lies in several important aspects. First, it provides one of the few integrated analyses of environmental quality, renewable energy transition, economic growth, healthcare expenditure, and mortality dynamics within a unified life expectancy framework for GCC countries. Second, unlike conventional static panel approaches, this study explicitly accounts for cross-sectional dependence, slope heterogeneity, endogeneity, and dynamic persistence through the application of second-generation panel techniques and the System Generalized Method of Moments (System GMM) estimator. Third, the study contributes to the public health and environmental health literature by highlighting the role of clean energy transition and environmental sustainability in improving longevity outcomes within resource-dependent economies.

This gap is particularly relevant given ongoing economic diversification and sustainability initiatives, such as Saudi Vision 2030 and the UAE Energy Strategy 2050, which explicitly seek to balance economic growth with environmental sustainability. Assessing whether these structural transformations translate into measurable improvements in population health is therefore of considerable policy importance. These policy initiatives increasingly emphasize renewable energy deployment, green urban development, environmental regulation, and preventive healthcare systems as central pillars of sustainable development and public health improvement. However, empirical evidence regarding the effectiveness of these transformations in improving life expectancy remains limited in the GCC context.

By employing a dynamic panel-data approach, specifically the System Generalized Method of Moments (System GMM), this study provides robust evidence that accounts for endogeneity, unobserved heterogeneity, and dynamic persistence in life expectancy. The analysis further incorporates cross-sectional dependence tests, slope heterogeneity analysis, second-generation panel unit root tests, cointegration procedures, Granger non-causality analysis, and robustness checks based on Driscoll–Kraay standard errors to ensure reliable statistical inference. The findings offer policy-relevant insights into the relative roles of clean energy adoption, healthcare expenditure, and environmental regulation in promoting longevity in resource-dependent economies.

The remainder of the paper is organized as follows. Section 2 reviews the relevant theoretical and empirical literature. Section 3 outlines the econometric methodology. Section 4 describes the data and variables. Section 5 presents and discusses the empirical results. Section 6 concludes with policy implications, limitations, and recommendations for improving life expectancy in the GCC countries and for guiding future research.

## 2. Literature Review

Life expectancy is a critical indicator of public health performance, economic development, and environmental sustainability. A substantial body of research has examined its key determinants, including mortality rates, healthcare expenditure, economic growth, and environmental conditions. Recent public health and environmental health studies increasingly emphasize the importance of integrating environmental sustainability, renewable energy transition, and healthcare investment in explaining long-term health outcomes. This section synthesizes recent empirical evidence, with particular emphasis on studies relevant to the GCC context. To improve clarity and organization, the literature is categorized into six major thematic dimensions: theoretical foundations, mortality dynamics, renewable energy and environmental health, economic growth, healthcare expenditure, and environmental degradation.

### 2.1. Theoretical Motivation for Empirical Analysis

The empirical investigation of life expectancy determinants is grounded in several complementary theoretical frameworks. The Preston Curve [2] illustrates the relationship between income and life expectancy, emphasizing that economic growth initially generates substantial health gains, while non-income factors become increasingly important at higher development levels. This perspective is consistent with Demographic Transition Theory, which describes the shift from high mortality and fertility to lower rates as economies develop, accompanied by a rising burden of non-communicable diseases [8,9].

In parallel, the Environmental Kuznets Curve (EKC) hypothesis posits that environmental degradation initially worsens with economic growth but eventually declines as cleaner technologies and stricter regulations are adopted [10]. This framework is particularly relevant for GCC economies, where rapid industrialization has generated significant environmental externalities with direct public health consequences. Human Capital Theory [11] further emphasizes that investments in health and education enhance productivity and long-term growth, while the Social Determinants of Health (SDH) framework [12] highlights the role of socioeconomic, environmental, and institutional factors in shaping health outcomes.

In the context of environmental health, these theoretical perspectives suggest that sustainable economic development requires balancing industrial growth with environmental protection and healthcare accessibility. The increasing integration of renewable energy into national development strategies is therefore expected to generate both environmental and public health benefits through reductions in pollution exposure and improvements in living conditions. Together, these frameworks justify the joint examination of mortality, renewable energy, economic growth, healthcare expenditure, and CO_2_ emissions in the GCC context.

### 2.2. Death Rate and Life Expectancy

The negative relationship between mortality rates and life expectancy is well established. Preston (1975) [2] demonstrated a strong inverse association, particularly in lower-income settings, while more recent studies confirm that reductions in crude death rates—driven by medical progress and public health interventions—are central to longevity improvements [13,14]. Evidence from the GCC region further suggests that governance and political stability indirectly influence health outcomes through their effects on economic performance and public service provision [15].

Within the GCC region, the epidemiological transition has reduced infectious disease mortality but increased the prevalence of NCDs, including cardiovascular disease, diabetes, and cancer [16]. High obesity rates and traffic-related fatalities remain major contributors to mortality, particularly in Saudi Arabia and the UAE [4,8]. These patterns underscore the importance of preventive healthcare policies and road safety regulations for improving life expectancy. Moreover, increasing urbanization, sedentary lifestyles, and environmental stressors have intensified public health concerns in GCC economies, reinforcing the need for integrated healthcare and environmental strategies.

### 2.3. Renewable Energy Consumption and Health Outcomes

Environmental quality is a key determinant of population health, with air pollution emerging as a major driver of premature mortality [17]. Exposure to pollutants from fossil fuel combustion increases the incidence of respiratory and cardiovascular diseases [5,18]. Empirical evidence indicates that transitions toward renewable energy reduce pollution-related mortality and improve overall health outcomes [6,19]. Cross-country evidence also shows that economic growth and air pollution jointly influence life expectancy outcomes [20]. Recent Gulf Cooperation Council-focused studies confirm that lower energy intensity and reduced carbon dioxide emissions enhance environmental quality and economic performance [21]. Recent studies further demonstrate that renewable energy expansion generates substantial health co-benefits by reducing PM_2.5_ exposure, greenhouse gas emissions, and pollution-related mortality [22,23]. In addition, Karimi Alavijeh et al. (2024) [24] show that renewable energy consumption positively influences life expectancy in advanced economies, reinforcing the public-health benefits of clean energy transition policies.

Although the GCC region has historically relied on hydrocarbons, renewable energy initiatives under Saudi Vision 2030 and the UAE Energy Strategy 2050 aim to mitigate environmental and health risks [25]. Empirical findings suggest that increased renewable energy consumption improves air quality and contributes to longer life expectancy [26], reinforcing the relevance of clean energy transitions for public health in the region. Recent GCC sustainability research further emphasizes that successful green-energy transition in Saudi Arabia requires stronger institutional coordination, sustainable urban planning, and effective environmental governance frameworks [25].

From a public health perspective, renewable energy adoption contributes to lower concentrations of airborne pollutants such as PM_2.5_ and greenhouse gas emissions, thereby reducing the prevalence of respiratory illnesses, cardiovascular diseases, and pollution-related mortality. Consequently, renewable energy transition should be viewed not only as an environmental objective but also as a strategic public health intervention capable of improving long-term population well-being [27]. This interpretation is supported by recent environmental-health literature emphasizing that renewable energy deployment can significantly reduce healthcare burdens associated with air pollution and climate-related environmental stress [23].

### 2.4. Economic Growth and Life Expectancy

The relationship between economic growth and life expectancy is complex and context-dependent [20]. While higher GDP per capita improves access to healthcare, nutrition, and living standards [28], economic growth alone does not guarantee improved health outcomes [1]. In the GCC countries, economic expansion must be accompanied by environmental sustainability and effective public investment to generate durable health benefits [29]. Persistent disparities in healthcare access, particularly for expatriate workers, highlight the need for inclusive health policies [30].

Recent studies further argue that the health benefits of economic growth increasingly depend on institutional quality, healthcare accessibility, environmental sustainability, and social inclusion rather than income growth alone. In highly resource-dependent economies, rapid industrialization and urbanization may simultaneously generate environmental externalities that offset part of the positive health effects associated with higher income levels. Recent evidence from sustainability and environmental-health research also indicates that climate resilience, environmental governance, and healthcare system efficiency increasingly shape the relationship between economic development and public health outcomes in emerging economies [31,32,33].

### 2.5. Government Health Expenditure and Life Expectancy

Public healthcare spending is a central determinant of longevity. Increased government health expenditure has been consistently linked to higher life expectancy through improved service quality and access [34,35]. In the GCC countries, rising health expenditure has supported advanced medical infrastructure and digital health innovations, particularly in Saudi Arabia and the UAE [36]. However, workforce shortages and reliance on foreign medical professionals remain challenges, reinforcing the importance of sustained investment in preventive care and human capital development [13,37,38]. Recent healthcare sustainability studies further emphasize that resilient healthcare systems increasingly depend on digital transformation, telemedicine integration, climate-adaptive healthcare infrastructure, and environmentally sustainable healthcare practices [31].

In addition, recent healthcare reforms across GCC countries increasingly emphasize preventive healthcare systems, telemedicine, AI-driven healthcare technologies, and digital health infrastructure to improve healthcare efficiency and long-term population health outcomes. These developments highlight the strategic role of healthcare investment in supporting sustainable public health systems within rapidly transforming economies. Recent evidence also suggests that integrating green healthcare practices, energy-efficient medical facilities, and AI-assisted healthcare systems can significantly improve healthcare sustainability and long-term public health resilience in rapidly urbanizing economies [32].

### 2.6. CO_2_ Emissions and Public Health

Environmental pollution, particularly CO_2_ emissions, poses significant risks to public health. High pollution levels are associated with respiratory and cardiovascular diseases, increased morbidity, and reduced life expectancy [5,19,39]. Rapid urbanization and industrialization have intensified these risks in major GCC cities, such as Riyadh, Dubai, and Doha [40]. Sustainable energy policies, green urban planning, and clean transportation systems are therefore essential for mitigating pollution-related health impacts. Recent environmental-health studies confirm that reductions in energy-related emissions can generate substantial public-health gains through improved air quality and lower pollution-related mortality rates [22,23].

The recent environmental health literature further emphasizes that long-term exposure to pollution and climate-related environmental stress can increase healthcare burdens, weaken population resilience, and reduce quality of life. Consequently, stricter environmental regulation, cleaner production systems, and renewable energy deployment are increasingly recognized as essential components of sustainable public health policy. Recent GCC-focused sustainability research additionally highlights the importance of integrating environmental governance, renewable energy deployment, and sustainable urban planning to mitigate climate-related health risks in the region [25].

Overall, the literature indicates that mortality, environmental degradation, and healthcare access play decisive roles in shaping life expectancy, while renewable energy adoption, public health expenditure, and inclusive economic growth contribute positively. These insights motivate the empirical strategy adopted in this study and underscore the importance of integrated policy approaches for enhancing longevity in the GCC countries. However, despite the growing literature, empirical evidence jointly examining environmental sustainability, renewable energy transition, healthcare expenditure, and life expectancy within a dynamic panel-data framework remains limited in the GCC context. This study therefore contributes to the literature by providing an integrated analysis that explicitly accounts for cross-sectional dependence, slope heterogeneity, endogeneity, and dynamic persistence in health outcomes.

A concise synthesis of the main empirical studies related to environmental quality, renewable energy, and life expectancy is provided in Appendix A Table A1.

## 3. Methodology

This study adopts a comprehensive dynamic panel-data framework designed to rigorously examine the determinants of life expectancy in the GCC countries. The methodology integrates cross-sectional dependence analysis, slope heterogeneity testing, panel unit root and cointegration procedures, dynamic panel estimation, and causality analysis, ensuring robust inference in the presence of interdependence, heterogeneity, and endogeneity. The methodological framework is specifically designed to address the econometric challenges commonly observed in GCC panel data, including strong regional interdependence, heterogeneous country dynamics, endogeneity, and dynamic persistence in health outcomes. Furthermore, the approach is aligned with the environmental health and public health objectives of the study by jointly modeling the interactions among environmental quality, renewable energy transition, economic performance, and healthcare investment.

### 3.1. Cross-Sectional Dependence Tests

Cross-sectional dependence (CD) tests are employed to detect contemporaneous correlations across cross-sectional units, which may arise from unobserved common factors, regional spillovers, or shared economic and environmental shocks. Ignoring cross-sectional dependence can lead to biased standard errors and misleading statistical inference. This issue is particularly relevant in the GCC context because member countries share similar economic structures, environmental conditions, energy markets, and regional policy frameworks. Consequently, economic or environmental shocks affecting one country may simultaneously influence neighboring economies.

To address this issue, the study applies three complementary tests. First, the Pesaran CD test [41] is based on the average pairwise correlation coefficients of residuals obtained from individual regressions. Second, the Friedman test [42] provides a non-parametric alternative suitable for panels with a small number of time periods. Third, the Frees test [43] relies on the sum of squared cross-correlations, offering additional robustness. The joint application of these tests ensures reliable detection of cross-sectional dependence in the panel.

### 3.2. Slope Heterogeneity Test

Given the structural and institutional differences across GCC countries, the assumption of homogeneous slope coefficients may be overly restrictive. Therefore, the study employs the Pesaran and Yamagata (2008) [44] slope heterogeneity test, which evaluates whether individual-specific slope coefficients differ significantly from pooled estimates. Rejecting slope homogeneity justifies the use of econometric techniques that accommodate heterogeneous dynamics across countries. Such heterogeneity is expected because GCC countries differ substantially in terms of renewable energy transition progress, healthcare infrastructure, demographic composition, environmental regulation, and economic diversification strategies.

### 3.3. Panel Unit Root Tests

To assess the stationarity properties of the variables, second-generation panel unit root tests are applied, as they explicitly account for cross-sectional dependence. In particular, the Cross-sectionally Augmented Im–Pesaran–Shin (CIPS) test and the Cross-sectionally Augmented Dickey–Fuller (CADF) test proposed by Pesaran (2007) [45] are employed. These tests augment standard unit root regressions with cross-sectional averages, thereby controlling unobserved common factors.

For comparison and completeness, traditional first-generation tests, including the Levin–Lin–Chu (LLC) [46] test and the Im–Pesaran–Shin (IPS) [47] test, are also reported. Establishing the order of integration is essential to avoid spurious regression results and to guide the subsequent cointegration and dynamic estimation procedures. The use of second-generation panel unit root tests is particularly important in this study because first-generation procedures may produce biased results when cross-sectional dependence is present.

### 3.4. Cointegration Tests

To examine the existence of long-run equilibrium relationships among the variables, multiple panel cointegration tests are implemented. The Kao (1999) [48] test adopts a residual-based approach derived from a pooled regression framework. In contrast, the Pedroni (1999, 2004) [49,50] tests allow for heterogeneous cointegrating vectors across cross-sectional units, making them particularly suitable for heterogeneous panels. Additionally, the Westerlund (2007) [51] error-correction-based test is employed, which explicitly accounts for cross-sectional dependence and focuses on short-run adjustments toward long-run equilibrium. Using multiple cointegration tests enhances the robustness of the inference regarding long-run relationships.

Given the relatively small number of cross-sectional units (N = 6) and the strong evidence of cross-sectional dependence, mixed cointegration results may emerge due to the limited statistical power of residual-based panel cointegration procedures. Therefore, the analysis interprets cointegration evidence cautiously and complements these tests with dynamic panel-data estimation techniques that do not strictly rely on long-run equilibrium assumptions.

### 3.5. Dynamic Panel Data Estimation

To model the dynamic behavior of life expectancy and address potential endogeneity, the study employs the Arellano–Bond (1991) [52] Generalized Method of Moments (GMM) estimator. This estimator is particularly appropriate in dynamic panels with a relatively small-time dimension and potential endogeneity arising from lagged dependent variables and simultaneity. The use of System GMM is additionally motivated by the possibility of reverse causality between health outcomes and explanatory variables such as economic growth, healthcare expenditure, and environmental quality.

The baseline dynamic panel model is specified as:

(1) $$y_{it}=\alpha y_{i,t-1}+x_{it}\beta +\eta_{i}+\varepsilon_{it}$$

where

$y_{it}$ denotes the dependent variable for country *i* at time *t*;

$y_{i,t-1}$ represents the lagged dependent variable capturing dynamic persistence;

$x_{it}$ is a vector of explanatory variables;

β is the corresponding vector of coefficients;

$\eta_{i}$ captures unobserved country-specific fixed effects;

$\varepsilon_{it}$ is the idiosyncratic error term, assumed to be serially uncorrelated.

To eliminate the unobserved fixed effects, the model is first-differenced:

(2) $${\Delta y}_{it}=\alpha {\Delta y}_{i,t-1}+{\Delta x}_{it}\beta +{\Delta \varepsilon }_{it}$$

where Δ denotes the first-difference operator.

However, the differenced lagged dependent variable $\Delta y_{i,t-1}$ is correlated with the differenced error term $\Delta \varepsilon_{it}$ , generating an endogeneity problem. The Arellano–Bond estimator resolves this issue by using appropriately lagged levels of the dependent variable and selected regressors as internal instruments, under the assumption of no serial correlation in the idiosyncratic errors.

To strengthen identification, instrument validity is rigorously assessed using Hansen and Sargan tests of over-identifying restrictions. Furthermore, Arellano–Bond tests for first-order and second-order serial correlation are conducted to confirm the absence of second-order autocorrelation, which is crucial for instrument validity. These diagnostic procedures ensure that the estimated coefficients are both consistent and reliable. Although the Hansen and Sargan *p*-values are relatively close to conventional significance thresholds, they remain within acceptable ranges, supporting the overall validity of the selected instruments and the robustness of the estimation strategy.

### 3.6. Granger Non-Causality Test

To explore the direction of causality among the variables, the study applies the Granger non-causality test developed by Juodis, Karavias, and Sarafidis (2021) [53]. This approach is well-suited to heterogeneous panels and explicitly accounts for cross-sectional dependence, addressing key limitations of traditional panel Granger causality tests. The test enables the identification of short-run predictive relationships while allowing for heterogeneous dynamic responses across countries.

Importantly, the Granger causality framework captures predictive short-run dynamic relationships rather than long-run structural effects. Consequently, the causality results should be interpreted as indicators of temporal predictive relationships rather than evidence of stable long-run equilibrium interactions.

### 3.7. Robustness Checks

Given the small number of cross-sectional units (six GCC countries) and the strong evidence of cross-sectional dependence, additional non-GMM robustness checks were also implemented. In particular, the study employs fixed-effects estimation with Driscoll–Kraay standard errors, which explicitly correct for heteroskedasticity, serial correlation, and cross-sectional dependence. This approach provides reliable inference in panels characterized by common shocks and regional spillovers.

The Driscoll–Kraay estimator is used as a conservative robustness procedure because it remains robust in the presence of strong cross-sectional dependence and heterogeneous error structures. In addition, robustness checks are intended to verify that the principal findings are not driven by a single estimation technique or by potential weaknesses associated with dynamic panel instrumentation in small-N settings.

The combined use of GMM-based and non-GMM estimators ensures that the main findings are not driven by a specific estimation technique or by potential weaknesses associated with dynamic panel instrumentation in small-N settings. Given the relatively small cross-sectional dimension of the GCC sample, the GMM findings should be interpreted cautiously despite the robustness checks performed.

Overall, this methodological framework provides a rigorous and internally consistent approach to analyzing the dynamic determinants of life expectancy in the GCC countries. By jointly addressing cross-sectional dependence, slope heterogeneity, non-stationarity, endogeneity, and causality, the study ensures robust and policy-relevant empirical findings. The methodology therefore provides a reliable framework for examining the interconnected relationships among environmental sustainability, renewable energy transition, healthcare investment, and public health outcomes within GCC economies.

## 4. Data

This study employs an unbalanced panel dataset covering six Gulf Cooperation Council countries—Bahrain, Kuwait, Oman, Qatar, Saudi Arabia, and the United Arab Emirates—over the period 2000–2023. All data are obtained from the World Bank’s World Development Indicators, ensuring international comparability, consistency, and reliability. The dataset is designed to examine the interrelationships between economic development, environmental factors, and health outcomes in the GCC region. The selected sample period captures major economic, environmental, and healthcare transformations in the GCC countries, including rapid urbanization, economic diversification strategies, renewable energy transition initiatives, and healthcare system reforms.

The analysis focuses on life expectancy and its association with renewable energy consumption, CO_2_ emissions, economic growth, government health expenditure, and mortality rates. Using a long-time horizon allows the study to capture both short-term fluctuations and longer-term structural dynamics in the region’s health outcomes. The selected variables are grounded in the theoretical and empirical literature linking environmental sustainability, healthcare systems, economic performance, and public health outcomes. In particular, the variable selection reflects the multidimensional nature of life expectancy determinants within resource-dependent and rapidly urbanizing economies.

**Figure 1:**
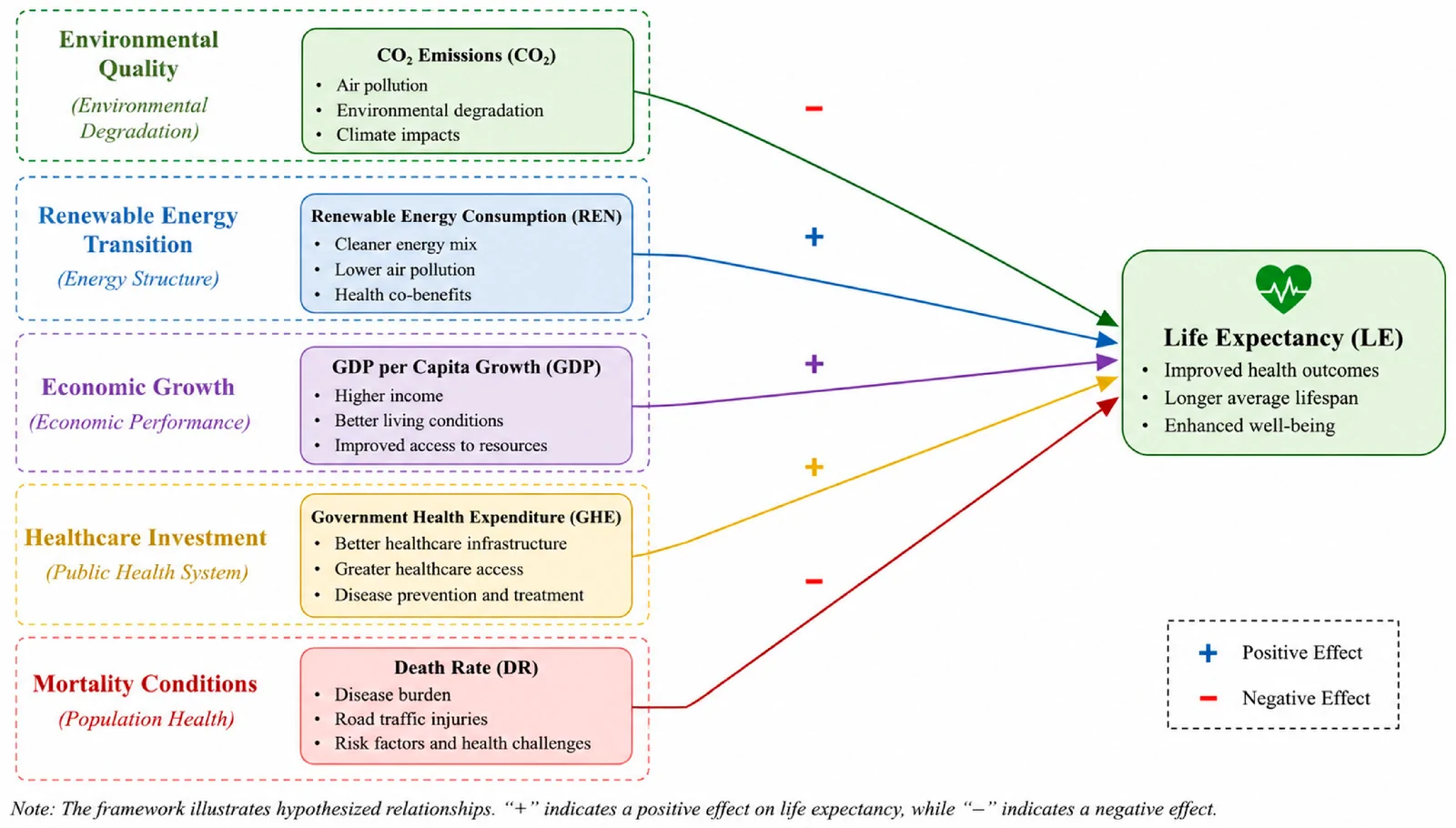
Conceptual Framework: Determinants of Life Expectancy in GCC Countries.

Figure 1 presents the conceptual framework of the study, illustrating the hypothesized relationships among environmental quality, renewable energy transition, economic growth, healthcare expenditure, mortality conditions, and life expectancy in the GCC countries. The framework assumes that renewable energy adoption and healthcare investment improve life expectancy, whereas environmental degradation and mortality pressures adversely affect public health outcomes.

Table 1 presents the definition and measurement of the variables employed in the empirical analysis. For clarity, the main variables are abbreviated as follows: life expectancy at birth (LEB), death rate (DR), renewable energy consumption (REN), gross domestic product per capita growth (GDP), carbon dioxide emissions (CO_2_), and government health expenditure (GHE). These abbreviations are used mainly in tables and econometric results.

The dependent variable, life expectancy at birth, measures the average number of years a newborn is expected to live under prevailing mortality conditions. The death rate, expressed as crude deaths per 1000 people, captures overall mortality conditions and provides an additional health-related control. Renewable energy consumption reflects the degree of transition toward cleaner energy sources and its potential health co-benefits. Gross domestic product per capita growth captures macroeconomic performance and improvements in living standards. Carbon dioxide emissions proxy environmental degradation associated with economic activity, while government health expenditure measures public investment in healthcare systems and preventive services.

The inclusion of renewable energy and CO_2_ emissions is particularly important from an environmental health perspective because pollution exposure and fossil-fuel dependence are strongly associated with respiratory diseases, cardiovascular disorders, and reduced life expectancy. Similarly, government health expenditure captures the capacity of healthcare systems to provide preventive care, medical treatment, and public health services capable of improving longevity outcomes.

Table 2 reports the pairwise correlation matrix among the variables.

The correlation matrix reveals several notable patterns. Life expectancy (LEB) is strongly and negatively correlated with the death rate (DR), consistent with standard demographic theory. LEB also exhibits a moderate positive correlation with CO_2_ emissions and a weaker positive correlation with government health expenditure, suggesting that in the GCC context, higher emissions and public spending may coincide with broader development processes that raise life expectancy.

At the same time, death rates are negatively correlated with CO_2_ emissions and positively correlated with health expenditure, indicating complex interactions between environmental conditions and health policy responses. Renewable energy consumption displays relatively weak correlations with most variables, reflecting its still-limited penetration in the GCC energy mix. GDP per capita growth shows weak correlations across the board, suggesting that short-term growth fluctuations may not translate directly into immediate health improvements. Overall, the correlation analysis highlights the multifaceted and non-linear relationships among economic, environmental, and health variables in the region.

Importantly, the positive unconditional correlation between CO_2_ emissions and life expectancy should not be interpreted as evidence that pollution improves health outcomes. Rather, this relationship likely reflects development-stage effects, whereby higher industrialization and income levels simultaneously increase emissions and improve healthcare infrastructure and living standards. The dynamic regression analysis therefore provides a more reliable assessment of the net conditional impact of environmental degradation on life expectancy after controlling for multiple economic and health-related factors.

Table 3 presents descriptive statistics for all variables used in the analysis.

The descriptive statistics indicate that life expectancy in the GCC countries is relatively stable over the sample period, reflecting the region’s advanced healthcare infrastructure. Death rates exhibit greater variability, capturing demographic differences and temporal fluctuations across countries. Renewable energy consumption remains very low on average, with several observations indicating no renewable adoption, underscoring the continued dominance of fossil fuels.

GDP per capita growth is highly volatile, reflecting exposure to oil price cycles and global economic shocks. CO_2_ emissions per capita are comparatively high, highlighting the energy-intensive structure of GCC economies and substantial cross-country heterogeneity. Government health expenditure shows moderate variation, suggesting differences in fiscal commitment to healthcare across countries and over time.

The relatively low level of renewable energy penetration observed in the descriptive statistics confirms that GCC economies remain highly dependent on hydrocarbon-based energy systems despite recent sustainability initiatives. Similarly, the high level of CO_2_ emissions reflects the environmental pressures associated with rapid urbanization, industrialization, and fossil-fuel-intensive economic structures.

Overall, the data underscore the structural characteristics of the GCC—high income, high emissions, limited renewable penetration, and substantial public health investment—providing a suitable empirical basis for analyzing the determinants of life expectancy. These characteristics further justify the importance of examining the interconnected relationships among environmental sustainability, renewable energy transition, healthcare investment, and public health outcomes within the GCC context.

## 5. Results

The empirical analysis begins by examining cross-sectional dependence (CD), a crucial issue in panel-data settings involving countries that may be exposed to common economic, environmental, and institutional shocks. Ignoring such dependence can lead to biased inference and invalid standard errors. This issue is particularly relevant in the GCC context because member countries are highly interconnected through common oil markets, regional policy coordination, similar environmental conditions, and shared economic structures.

### 5.1. Cross-Sectional Dependence

Table 4 reports the results of the cross-sectional dependence tests.

The CD test results provide strong evidence of cross-sectional dependence across all variables, with test statistics significant at conventional levels. The null hypothesis of cross-sectional independence is therefore decisively rejected. Among the variables, CO_2_ emissions exhibit the strongest degree of dependence, reflecting shared environmental and energy-related dynamics across GCC countries.

The Pesaran and Friedman tests further confirm the presence of strong cross-sectional dependence, while the Frees statistic exceeds its critical value, reinforcing this conclusion despite the absence of an associated *p*-value. These findings indicate substantial interdependence among GCC countries, likely driven by common economic structures, energy markets, environmental conditions, and regional policy frameworks.

The presence of significant cross-sectional dependence implies that shocks affecting one GCC country may rapidly spill over to neighboring economies. Consequently, the use of econometric approaches capable of controlling for cross-sectional dependence is necessary to avoid biased standard errors and misleading statistical inference. This finding further justifies the application of second-generation panel procedures and Driscoll–Kraay robustness estimators in the subsequent analysis.

### 5.2. Slope Heterogeneity

Table 5 presents the results of the Pesaran and Yamagata (2008) [44] slope heterogeneity test.

The null hypothesis of slope homogeneity is rejected at the 1% significance level, indicating that the impact of explanatory variables on life expectancy differs across GCC countries. This result justifies the use of estimation techniques that account for heterogeneity and reinforces the appropriateness of the dynamic panel GMM framework adopted in this study.

The rejection of slope homogeneity is expected given the structural differences among GCC countries in terms of healthcare systems, environmental regulation, renewable energy transition progress, demographic characteristics, and economic diversification strategies. These heterogeneous country-specific conditions imply that environmental and economic variables may influence life expectancy differently across GCC economies.

### 5.3. Panel Unit Root Tests

To ensure the validity of the econometric analysis, panel unit root tests accounting for cross-sectional dependence are conducted. The results of the Pesaran CIPS and CADF tests are reported in Table 6.

The results indicate that life expectancy (LEB), death rate (DR), renewable energy consumption (REN), CO_2_ emissions (CO_2_), and government health expenditure (GHE) are non-stationary in levels, while GDP per capita growth is stationary at level. After first difference, all variables become stationary, confirming that they are integrated of order I(0) or I(1) and suitable for dynamic panel estimation without risk of spurious regression.

The use of second-generation panel unit root tests is particularly important because first-generation tests may produce biased results in the presence of strong cross-sectional dependence. The stationarity findings therefore support the reliability of the subsequent dynamic panel-data estimation procedures.

### 5.4. Cointegration Analysis

Table 7 reports the results of the panel cointegration tests.

The cointegration tests provide mixed and inconclusive evidence regarding the existence of a long-run equilibrium relationship among the variables. Specifically, the Pedroni test indicates the presence of cointegration in at least one specification, as reflected by the statistically significant modified Phillips–Perron statistic. In contrast, the Kao test statistics are largely insignificant, and the Westerlund variance ratio test fails to reject the null hypothesis of no cointegration.

These divergent findings are not uncommon in panel-data settings characterized by small cross-sectional dimensions (N = 6), cross-sectional dependence, and slope heterogeneity, all of which are strongly present in the GCC sample. Under such conditions, residual-based and error-correction-based cointegration tests may exhibit reduced power and yield conflicting results.

Moreover, dynamic panel estimators such as System GMM are widely used in the absence of confirmed cointegration, particularly when the focus is on conditional relationships rather than long-run equilibrium coefficients.

Importantly, the absence of consistent cointegration evidence does not invalidate empirical analysis, as the chosen estimation strategy does not rely on strict long-run equilibrium assumptions. Instead, the study employs a dynamic panel-data framework that explicitly models short-run adjustments and dynamic interactions among variables.

Given these considerations, the analysis proceeds using the System Generalized Method of Moments (System GMM), which is particularly suitable for panels with potential endogeneity, heterogeneity, and dynamic persistence. System GMM provides consistent and efficient estimates regardless of whether a stable cointegration relationship exists, making it an appropriate and robust methodological choice in the present context.

The mixed cointegration findings should therefore be interpreted cautiously rather than viewed as contradictory evidence. In small-N panels such as the GCC sample, the statistical power of panel cointegration procedures may be limited, particularly under strong cross-sectional dependence. Consequently, the empirical analysis emphasizes dynamic conditional relationships and short-run adjustments rather than strict long-run equilibrium interpretations.

### 5.5. Dynamic Panel GMM Estimation

Table 8 presents the results of the one-step System GMM estimation.

The results provide several important insights into the determinants of life expectancy in the GCC countries. The lagged change in life expectancy is statistically insignificant, suggesting limited persistence in short-run life expectancy dynamics. This finding indicates that recent changes in life expectancy are primarily influenced by contemporaneous environmental, healthcare, and economic conditions rather than by historical inertia.

The change in death rate exhibits a negative and highly significant effect on life expectancy, confirming that higher mortality directly reduces longevity. The change in renewable energy consumption shows a positive and statistically significant coefficient, indicating that clean energy adoption contributes to improved health outcomes, likely through reduced environmental pollution and better air quality.

From an environmental health perspective, the positive effect of renewable energy consumption suggests that cleaner energy systems may reduce pollution-related respiratory and cardiovascular diseases, thereby contributing to improved public health outcomes and longer life expectancy.

GDP per capita growth is positively associated with life expectancy, but its economic magnitude is extremely small, implying that economic growth alone has a limited direct effect on longevity in high-income, resource-based economies such as the GCC countries. This highlights the importance of complementary social, environmental, and health policies.

CO_2_ emissions exert a negative and statistically significant effect on life expectancy, underscoring the adverse health consequences of environmental degradation. Government health expenditure has a positive and significant impact, confirming that public investment in healthcare plays a crucial role in extending life expectancy.

The apparent contrast between the positive correlation of CO_2_ with life expectancy (Table 2) and its negative regression coefficient reflects development-stage effects: emissions may initially rise alongside industrialization and income growth, but their long-run health impacts are detrimental once pollution thresholds are exceeded.

More specifically, the positive unconditional correlation observed in the descriptive statistics likely captures broader development effects associated with industrialization and income growth. However, once economic and healthcare factors are controlled for in the dynamic regression framework, the net conditional effect of CO_2_ emissions becomes negative, reflecting the harmful environmental health consequences of pollution exposure.

### 5.6. Diagnostic Tests

Table 9 reports the diagnostic tests for the System GMM estimation.

The diagnostic results confirm the validity of the model specification. The Arellano–Bond AR(1) test is significant, as expected in first-differenced models, while the AR(2) test is insignificant, indicating no second-order serial correlation. Both the Hansen and Sargan tests fail to reject the null hypothesis of instrument validity, providing acceptable evidence that the instruments are appropriate. Although the *p*-values are close to conventional thresholds, they remain within acceptable bounds, suggesting cautious but valid inference.

The Hansen and Sargan statistics therefore support the overall reliability of the dynamic panel instrumentation strategy and indicate no serious evidence of over-identification problems or instrument invalidity.

### 5.7. Granger Non-Causality Analysis

Table 10 presents the results of the Juodis, Karavias, and Sarafidis (2021) [53] Granger non-causality test.

The HPJ Wald test strongly rejects the null hypothesis of joint non-causality, indicating the presence of dynamic causal relationships. Renewable energy consumption, CO_2_ emissions, and government health expenditure Granger-cause changes in life expectancy, while death rate and GDP growth do not exhibit significant short-run predictive power.

Importantly, the negative coefficients observed in the Granger causality framework reflect short-run dynamic adjustments rather than long-run structural effects. These results should therefore be interpreted as predictive relationships, not as contradictions to the positive long-run impacts identified in the GMM estimation.

Consequently, the Granger causality analysis should be interpreted as evidence of short-run predictive interactions among environmental, economic, and healthcare variables rather than as proof of stable long-run equilibrium relationships.

Overall, the results highlight the central roles of environmental quality and healthcare investment in shaping life expectancy in the GCC countries. Renewable energy adoption and public health expenditure emerge as key drivers of longevity, while CO_2_ emissions and mortality exert adverse effects. Economic growth alone plays a limited role unless accompanied by targeted health and environmental policies, underscoring the need for integrated development strategies in the region.

### 5.8. Robustness Checks

Table 11 reports the fixed-effects estimates with Driscoll–Kraay standard errors, which control for heteroskedasticity, serial correlation, and cross-sectional dependence.

The lagged dependent variable is statistically insignificant, indicating limited short-run persistence in changes in life expectancy once country fixed effects and common shocks are accounted for. The change in death rate exhibits a negative and highly significant effect, confirming that mortality reduction is the most robust and immediate determinant of life expectancy in the GCC countries.

Renewable energy consumption, GDP per capita growth, and government health expenditure display positive coefficients, although they are not statistically significant at conventional levels under this conservative estimator. This suggests that their health benefits are likely to operate through longer-term or indirect channels rather than short-run effects. Carbon dioxide emissions are statistically insignificant once cross-sectional dependence is controlled for, reflecting the difficulty of isolating country-specific pollution effects in a highly integrated regional context.

The Driscoll–Kraay estimator is intentionally employed as a conservative robustness procedure because it explicitly corrects for heteroskedasticity, serial correlation, and cross-sectional dependence. The broad consistency between the System GMM and Driscoll–Kraay results therefore reinforces the robustness and reliability of the principal findings.

Overall, the Driscoll–Kraay results support the robustness of the main conclusions, highlighting mortality reduction as the dominant driver of life expectancy, with economic, environmental, and policy factors playing complementary roles. These robustness findings further confirm that the principal conclusions are not driven by a single estimation method or by potential weaknesses associated with small-sample dynamic panel instrumentation.

## 6. Discussion and Policy Implications

Life expectancy is a key indicator of public health and overall well-being, reflecting the combined effectiveness of healthcare systems, economic conditions, and environmental policies. A broad body of literature demonstrates that longevity is shaped by mortality rates, healthcare expenditure, economic performance, and environmental sustainability [1,2]. Recent studies confirm that environmental quality, renewable energy use, healthcare system resilience, and climate-related risks have become increasingly important determinants of population health and longevity [22,23,24]. The findings of this study are consistent with this literature while providing new evidence on the mechanisms shaping life expectancy in the Gulf Cooperation Council (GCC) countries. More specifically, the results contribute to the environmental health and public health literature by highlighting the interconnected roles of renewable energy transition, environmental quality, healthcare investment, and mortality reduction in improving longevity outcomes within resource-dependent economies.

The discussion is organized according to the main determinants examined in the empirical model: mortality conditions, renewable energy consumption, economic growth, government health expenditure, carbon dioxide emissions, and lagged life expectancy dynamics. This structure ensures that each interpretation and policy implication is directly linked to a specific empirical result. For instance, the negative effect of death rates motivates recommendations related to preventive healthcare, chronic disease management, and road safety, while the positive effect of renewable energy consumption supports policies promoting clean energy transition and pollution reduction. Similarly, the positive role of government health expenditure justifies recommendations related to healthcare access, equity, and system resilience.

Although the empirical analysis focuses mainly on measurable macroeconomic, environmental, and public health determinants, broader ethical considerations are also relevant. Policies aimed at improving life expectancy should consider not only aggregate health gains but also fairness, inclusiveness, environmental justice, and the protection of vulnerable groups. These ethical dimensions are particularly important in Gulf Cooperation Council countries, where demographic diversity and the presence of large expatriate populations may create unequal access to healthcare services and uneven exposure to environmental risks. Therefore, the implications discussed below should be interpreted from both an efficiency perspective and an equity-oriented public health perspective.

### 6.1. The Negative Impact of Death Rates on Life Expectancy

A central finding of this study is the negative and highly significant relationship between the death rate (DDR) and life expectancy. This result is both theoretically and empirically expected, as higher crude death rates directly reduce average lifespan [54]. The magnitude and statistical significance of the coefficient underscore the critical role of mortality reduction as a primary driver of longevity improvements, in line with the seminal contributions of Preston (1975) [2] and Oeppen and Vaupel (2002) [7].

Because the death rate captures overall population health conditions, increases in mortality driven by disease prevalence, limited healthcare access, or environmental stressors translate directly into lower life expectancy. The Preston Curve framework emphasizes that mortality reductions remain crucial for extending life expectancy even in relatively high-income settings when health risks persist.

In the GCC countries, mortality dynamics reflect an ongoing epidemiological transition characterized by declining infectious diseases and a rising burden of non-communicable diseases (NCDs), including cardiovascular diseases, diabetes, and cancer [55]. High obesity and diabetes prevalence place the GCC among the most affected regions globally, contributing substantially to preventable mortality. In addition, traffic-related fatalities—particularly in Saudi Arabia and the United Arab Emirates—remain a major public health challenge [4]. These patterns highlight the urgent need for strengthened preventive healthcare, improved road safety enforcement, and more effective chronic disease management policies across the region.

The findings therefore reinforce the importance of public health policies focused on preventive medicine, lifestyle awareness campaigns, early disease detection, and improved healthcare accessibility. Addressing chronic diseases and preventable mortality remains essential for achieving sustainable improvements in population health and life expectancy across GCC countries.

### 6.2. Renewable Energy Consumption and Health Benefits

A particularly important result is the positive and statistically significant effect of renewable energy consumption (REN) on life expectancy. This finding aligns with growing international evidence linking clean energy transitions to improved environmental quality and public health outcomes [56]. Recent empirical evidence shows that renewable energy expansion can generate substantial air quality and health co-benefits by reducing fossil-fuel-related emissions, fine particulate matter exposure, and pollution-related premature mortality [22,23]. Similarly, Karimi Alavijeh et al. (2024) [24] demonstrate that renewable energy plays a positive role in improving life expectancy in advanced economies, reinforcing the relevance of clean energy transition for public health outcomes.

Fossil fuel combustion is a major source of air pollution, contributing to respiratory illnesses, cardiovascular diseases, and premature mortality [39]. The positive association between renewable energy adoption and life expectancy suggests that shifting the energy mix toward cleaner sources generates direct health co-benefits, primarily through reductions in air pollution and environmental exposure. This result is consistent with previous studies showing that countries investing in renewable energy experience fewer pollution-related deaths and lower morbidity rates [6,57]. It is also consistent with recent environmental-health research showing that renewable energy policies can simultaneously reduce greenhouse gas emissions and improve public health by lowering exposure to PM_2.5_, SO_2_, NO_x_, and other harmful pollutants.

In the GCC region—where economic development has historically relied on oil and gas—urban air pollution levels in cities such as Riyadh, Dubai, and Doha are among the highest globally. Recent policy initiatives, including Saudi Vision 2030, the Saudi Green Initiative, and the UAE Energy Strategy 2050, aim to address these environmental challenges by accelerating renewable energy deployment. Recent GCC-focused research also emphasizes that Saudi Arabia’s green energy transition is increasingly embedded within Vision 2030 and requires coordinated policy frameworks linking energy diversification, environmental protection, economic transformation, and public welfare [25]. The results of this study indicate that such strategies not only enhance environmental sustainability but also contribute meaningfully to longer life expectancy, reinforcing the public health rationale for energy diversification.

From an environmental health perspective, renewable energy transition contributes to lower concentrations of harmful pollutants such as PM_2.5_ and greenhouse gas emissions, thereby reducing the prevalence of respiratory and cardiovascular diseases. Consequently, renewable energy policies should be viewed not only as environmental sustainability initiatives but also as long-term public health strategies capable of improving population well-being and reducing healthcare burdens.

### 6.3. Economic Growth and Life Expectancy

Economic growth, measured by GDP per capita growth, exhibits a statistically significant but economically modest positive effect on life expectancy. This finding is consistent with classical economic theory linking higher income levels to improved nutrition, healthcare access, and living conditions [28]. However, the small magnitude of the coefficient indicates that economic growth alone is not a dominant driver of longevity in the GCC context.

This result supports the argument advanced by Cutler et al. (2006) [1], who stress that the health benefits of economic growth depend critically on how resources are allocated and invested, particularly in public health systems. In high-income, resource-based economies such as the GCC countries, marginal gains from growth may be limited unless accompanied by targeted social, health, and environmental policies.

Despite high average income levels, substantial disparities in healthcare access persist, especially among low-income expatriate workers who constitute a large share of the GCC labor force [58]. While nationals generally benefit from comprehensive public healthcare coverage, expatriates often face financial and institutional barriers to medical services. These inequalities help explain why economic growth does not translate into proportionate gains in life expectancy, underscoring the importance of inclusive healthcare policies that extend access to all residents.

The findings therefore suggest that sustainable improvements in life expectancy require more than income expansion alone. Economic growth must be accompanied by environmental sustainability, equitable healthcare access, effective public health systems, and inclusive social policies to generate durable health benefits.

### 6.4. The Role of Government Health Expenditure

Government health expenditure (GHE) emerges as a strong and positive determinant of life expectancy, highlighting the central role of public investment in healthcare systems. Extensive empirical evidence shows that higher public health spending improves medical infrastructure, service availability, and disease prevention, thereby extending life expectancy [34,35]. Recent studies further emphasize that healthcare systems are increasingly expected to become more resilient, digitally enabled, and environmentally sustainable, especially as climate change and pollution impose additional burdens on public health systems [31,32].

In the GCC region, rising healthcare expenditure—particularly in Saudi Arabia and the UAE—has supported the expansion of advanced medical facilities, digital health platforms, and AI-driven healthcare solutions [36]. Nevertheless, challenges persist, including shortages of domestically trained medical professionals and continued reliance on foreign healthcare workers. Moreover, the growing burden of chronic diseases necessitates a strategic shift toward preventive and primary healthcare rather than treatment-focused systems. The findings therefore suggest that sustained investment in healthcare infrastructure, workforce development, and preventive health programs is essential for achieving further longevity gains in the region.

Recent healthcare reforms across GCC countries increasingly emphasize telemedicine, digital health systems, AI-assisted diagnostics, and preventive healthcare strategies. In line with the recent healthcare sustainability literature, GCC countries should also integrate green healthcare practices, energy-efficient hospital infrastructure, sustainable procurement, and climate-resilient health services into long-term public health planning [31,32]. These initiatives are particularly important for improving healthcare efficiency, expanding access to medical services, and strengthening long-term population health resilience.

### 6.5. CO_2_ Emissions and Life Expectancy

The analysis reveals a negative and statistically significant relationship between CO_2_ emissions and life expectancy, indicating that environmental degradation adversely affects population health in the GCC region. Although the estimated effect is relatively small, its statistical significance highlights the cumulative health burden associated with pollution exposure, consistent with global evidence linking air pollution to respiratory and cardiovascular diseases and premature mortality [5,39]. Recent environmental-health evidence confirms that reducing emissions from energy-related sectors can prevent premature deaths and generate substantial public health benefits through improvements in air quality [22,23].

Rapid industrialization, urban expansion, and high vehicle dependence have contributed to elevated pollution levels in major GCC cities, where environmental stress is further intensified by harsh climatic conditions. Even modest reductions in emissions can therefore generate meaningful public health benefits, particularly in densely populated urban areas. These results reinforce the need for integrated environmental policies, including renewable energy promotion, cleaner transportation systems, improved urban planning, and sustained investment in green technologies.

The findings further highlight the importance of environmental regulation and sustainable urban planning in reducing pollution-related health risks. This interpretation is consistent with recent GCC sustainability research showing that environmental sustainability depends not only on renewable energy expansion but also on institutional quality, digital transformation, urban planning, and effective implementation of national green transition strategies [25,59]. Policies promoting cleaner transportation systems, green infrastructure, and environmentally sustainable industrial practices are therefore essential for improving long-term environmental health outcomes in GCC countries.

### 6.6. The Lagged Life Expectancy Effect

The insignificance of the lagged life expectancy term suggests that recent changes in longevity are driven primarily by contemporaneous economic, environmental, and policy conditions rather than by historical trends. This finding aligns with Lutz et al. (2014) [60], who argue that life expectancy improvements increasingly depend on current policy choices, institutional quality, and environmental conditions, particularly in advanced and rapidly developing economies.

This result also suggests that current environmental and healthcare policies can generate relatively rapid improvements in population health outcomes, reinforcing the importance of timely policy intervention and effective institutional implementation.

### 6.7. Policy Implications

Taken together, the findings of this study yield several important policy implications for the Gulf Cooperation Council region. First, reducing mortality through preventive healthcare, strengthened road safety regulations, and improved chronic disease management should remain a top priority. This is particularly important given the growing burden of non-communicable diseases, including diabetes, obesity, cardiovascular disorders, and other lifestyle-related health risks. Expanding access to routine medical screening, early diagnosis, preventive care, and lifestyle-based interventions can substantially reduce avoidable mortality and improve population health outcomes.

Second, accelerating the transition toward renewable energy offers a dual dividend by enhancing environmental quality and improving public health. Policymakers should therefore scale up investments in solar and wind energy, enforce stricter air quality standards, and support carbon capture technologies, emissions monitoring systems, and clean transportation infrastructure. These measures can reduce pollution exposure, lower the incidence of respiratory and cardiovascular diseases, and generate simultaneous environmental and public health gains.

Third, economic growth alone is insufficient to generate substantial longevity gains unless it is accompanied by inclusive healthcare access and effective public spending. Accordingly, government health expenditure should be expanded with a strong emphasis on accessibility, equity, and preventive healthcare provision. This is especially important for low-income expatriate workers, who may face financial and institutional barriers to affordable medical services. Investments in telemedicine, artificial-intelligence-assisted healthcare solutions, and modern medical infrastructure can help bridge access gaps, particularly in underserved or remote areas.

Fourth, strengthening environmental regulation and reducing carbon dioxide emissions are essential for safeguarding long-term population health. These recommendations are consistent with recent evidence showing that renewable energy expansion, green healthcare systems, and climate-resilient public health policies can jointly improve environmental sustainability and health outcomes [23,25,31]. Evidence from low- and high-income countries further suggests that renewable energy consumption contributes significantly to reducing carbon emissions and promoting environmental sustainability, thereby generating important co-benefits for population health and longevity [61].

Road safety should also be treated as a central public health priority, particularly in countries such as Saudi Arabia and the United Arab Emirates, where traffic-related fatalities remain an important source of preventable mortality. Stronger enforcement of traffic regulations, improvements in road infrastructure, wider use of road safety technologies, and expansion of public transportation networks can significantly reduce accident-related deaths and injuries.

In addition, Gulf Cooperation Council governments should strengthen environmental monitoring systems, expand clean transportation infrastructure, support sustainable urban planning initiatives, and promote public awareness regarding pollution-related health risks. Sustainable urban planning should prioritize healthier living environments through walkable city designs, expanded green spaces, reduced vehicle dependence, and effective air pollution mitigation strategies. Addressing socioeconomic inequalities and healthcare disparities through inclusive public health policies is also essential for achieving durable improvements in life expectancy.

Ethical considerations should also guide the implementation of these policy recommendations. Improving life expectancy should not be limited to aggregate national gains but should also ensure fair access to healthcare services, preventive care, clean environments, and safe urban infrastructure for all population groups. Attention should be given to low-income expatriate workers, elderly populations, individuals with chronic diseases, and communities exposed to higher levels of pollution or traffic-related risks. From this perspective, environmental regulation, healthcare investment, renewable energy transition, and urban planning should be designed to reduce health inequalities rather than unintentionally widening them. Integrating ethical principles such as equity, inclusiveness, transparency, environmental justice, and intergenerational responsibility would strengthen the social legitimacy and long-term effectiveness of public health and environmental policies in the Gulf Cooperation Council region.

Greater coordination among environmental, healthcare, and economic institutions is essential for designing integrated development strategies capable of simultaneously improving environmental sustainability and public health outcomes. Such cross-sectoral coordination would help align clean energy transition, healthcare reform, urban planning, environmental regulation, and economic diversification within a unified public health-oriented development strategy.

Overall, the results underscore the need for integrated development strategies that jointly address health, environmental sustainability, and economic policy in order to achieve durable improvements in life expectancy across Gulf Cooperation Council countries.

## 7. Conclusions

This study provides a comprehensive empirical assessment of the key determinants of life expectancy in Gulf Cooperation Council countries, emphasizing the interconnected roles of mortality dynamics, environmental quality, energy structure, economic performance, and public health investment. The results demonstrate that higher death rates significantly reduce life expectancy, reaffirming the central importance of effective healthcare systems and targeted interventions addressing major mortality drivers, including chronic diseases and road traffic accidents.

Renewable energy consumption emerges as a positive and statistically significant contributor to longevity, indicating that reducing dependence on fossil fuels generates substantial public health benefits by lowering air pollution and the prevalence of pollution-related respiratory and cardiovascular diseases. Economic growth, although statistically significant, exerts only a modest effect on life expectancy, suggesting that income expansion alone is insufficient to produce meaningful longevity gains without complementary investments in healthcare, environmental protection, and social inclusion. In contrast, government health expenditure plays a decisive role in extending life expectancy, highlighting the importance of sustained public investment in healthcare infrastructure, workforce development, and disease prevention. Conversely, carbon dioxide emissions are negatively associated with life expectancy, underscoring the health costs of environmental degradation and the urgency of effective pollution control and emission reduction policies. This finding is consistent with recent evidence highlighting the adverse consequences of poor environmental quality on health and aging outcomes [62].

Despite these contributions, the study has several limitations that should be acknowledged. First, the analysis is limited to six Gulf Cooperation Council countries, resulting in a relatively small cross-sectional sample that may reduce the statistical power of certain panel procedures, particularly residual-based cointegration tests. Second, the study focuses primarily on macroeconomic and environmental indicators and does not incorporate additional public health variables such as PM_2.5_ exposure, healthcare quality indicators, climate variables, or behavioral health factors due to data limitations. Third, although the System Generalized Method of Moments estimator addresses important endogeneity concerns, the dynamic panel framework primarily captures short-run and medium-run relationships rather than strict long-run equilibrium effects.

Future research could extend the analysis by incorporating broader environmental health indicators, climate-related variables, healthcare quality measures, and non-linear modeling approaches. Additional studies focusing on individual Gulf Cooperation Council countries or comparing these economies with other emerging regions may also provide deeper insights into the environmental–health nexus and the role of renewable energy transition in improving public health outcomes. The present findings are also aligned with recent studies emphasizing that renewable energy transition represents a critical pathway for simultaneously improving environmental quality and reducing emissions-related health risks [60]. Furthermore, evidence from energy-transition economies suggests that sustained reductions in carbon emissions require coordinated policies promoting renewable energy deployment, technological innovation, and sustainable economic development [63].

Overall, the findings confirm that improving life expectancy in Gulf Cooperation Council countries requires an integrated development approach that links mortality reduction, clean energy transition, healthcare investment, and environmental protection. As these countries continue their structural transformation under initiatives such as Saudi Vision 2030 and the UAE Energy Strategy 2050, aligning public health strategies with environmental sustainability will be essential for achieving durable gains in population health and long-term sustainable development.

## Figures and Tables

**Table 1:** Data description.

Variable	Abbreviation
Life expectancy at birth, total (years) (ln)	LEB
Death rate, crude (per 1000 people) (ln)	DR
Renewable energy consumption (% of total final energy consumption)	REN
GDP per capita growth (annual %)	GDP
Carbon dioxide (CO_2_) emissions (total) (% change)	CO_2_
Domestic general government health expenditure (% of GDP) (ln)	GHE

**Table 2:** Correlation matrix.

Variable	LEB	DR	REN	GDP	CO_2_	GHE
LEB	1.0000					
DR	−0.6969	1.0000				
REN	0.1782	−0.2211	1.0000			
GDP	−0.1192	0.1068	−0.0155	1.0000		
CO_2_	0.5168	−0.4118	0.1097	−0.0756	1.0000	
GHE	0.1803	0.2227	0.0475	−0.2213	0.2631	1.0000

**Table 3:** Descriptive statistics.

Variable	Obs.	Mean	Std. Dev.	Min	Max
LEB	138	4.3473	0.0263	4.2831	4.4013
DR	138	0.6791	0.3602	−0.2294	1.3339
REN	132	0.0508	0.1368	0.0000	1.0000
GDP	144	−0.0510	4.7105	−11.8648	19.0059
CO_2_	138	212.3683	122.8261	48.2100	526.9659
GHE	132	0.8856	0.3440	0.1604	1.7353

**Table 4:** Test of Cross-Sectional Dependence results.

Cross-Sectional Dependence Test Results		
**Cross-Sectional Dependence (CD) test**		
**Variable**	**Statistic**	***p*-value**
LEB	24.1790	<0.001
DR	20.5580	<0.001
REN	4.1540	<0.001
GDP	3.1030	0.0020
CO_2_	21.4340	<0.001
GHE	12.3890	<0.001
**Cross-Sectional Dependence Test for Tests (Pesaran, Friedman, Frees)**		
**Test**	**Statistic**	***p*-value**
Pesaran’s Test	7.2970	<0.001
Friedman’s Test	66.6800	<0.001
Frees’ Test	1.6970	-

**Table 5:** Test for slope heterogeneity results.

	Slope Heterogeneity Test Results	
Test	Delta	*p*-Value
Unadjusted	9.6150	<0.001
Adjusted	11.6440	<0.001

**Table 6:** Panel Unit Root Test results.

Pesaran Panel Unit Root (CIPS) Test			Pesaran’s Cross-Sectional Augmented Dickey–Fuller (CADF) Test		
**Variables in levels**			**Variables in levels**		
**Variable**	**Statistic**	***p*-value**	**Variable**	**Statistic**	***p*-value**
LEB	−1.2330	0.1080	LEB	−1.5880	0.6920
DR	−0.6160	0.2690	DR	−0.8320	0.9930
CO_2_	−2.2530	0.0130	CO_2_	−2.4560	0.0390
REN	−2.1500	0.0170	REN	−0.5630	0.9990
GDP	−5.1060	<0.001	GDP	−4.3550	<0.001
GHE	−2.4900	0.0060	GHE	−2.5490	0.0230
**Variables after differencing**			**Variables after differencing**		
**Variable**	**Statistic**	***p*-value**	**Variable**	**Statistic**	***p*-value**
DLEB	−4.5610	<0.001	DLEB	−3.8020	<0.001
DDR	−3.6130	<0.001	DDR	−2.7000	0.0080
DCO_2_	−5.1670	<0.001	DCO_2_	−4.5410	<0.001
DREN	−4.3800	<0.001	DREN	−3.5620	<0.001
DGDP	−6.1900	<0.001	DGDP	−5.9840	<0.001
DGHE	−4.1170	<0.001	DGHE	−2.6360	0.0140

**Table 7:** Test of cointegration results.

	Cointegration Test Results.		
	Test Statistic	Value	*p*-Value
**Kao Test**	Modified Dickey–Fuller t-statistic	0.5282	0.2987
	Dickey–Fuller t-statistic	1.2906	0.0984
	Augmented Dickey–Fuller t-statistic	0.0219	0.4912
	Unadjusted Modified Dickey–Fuller t-statistic	0.9536	0.1701
	Unadjusted Dickey–Fuller t-statistic	1.7394	0.0410
**Pedroni Test**	Modified Phillips–Perron t-statistic	2.5771	0.0050
	Phillips–Perron t-statistic	0.5907	0.2774
	Augmented Dickey–Fuller t-statistic	1.1962	0.1158
**Westerlund Test**	Variance ratio	−0.3538	0.3617

**Table 8:** Dynamic panel-data estimation results.

Variable	Coefficient	Std. Err.	z	*p*-Value
L1.DLEB	0.0089	0.0356	0.2500	0.8020
DDR	−0.0881	0.0032	−27.8300	<0.001
DREN	0.0127	0.0043	2.9400	0.0030
GDP	0.0002	0.0001	4.6100	<0.001
CO_2_	−0.0001	<0.001	−2.0500	0.0410
GHE	0.0025	0.0007	3.4000	0.0010
_cons	0.0001	0.0008	0.1000	0.9220

**Table 9:** Diagnostic Tests for System GMM.

Test	Statistic	*p*-Value
Arellano–Bond Test for AR(1)	−1.9900	0.0460
Arellano–Bond Test for AR(2)	−0.1000	0.9160
Sargan test of overid. restrictions	52.1000	0.0910
Hansen test of overid. restrictions	49.7500	0.1080

**Table 10:** Granger Non-Causality Test Results.

**JKS Non-Causality Test for dleb (Life Expectancy)**				
**Test**	**Statistic**	***p*-value**		
HPJ Wald test	77.3607	<0.001		
**Half-Panel Jackknife Estimator (with lag 1)**				
**Variable**	**Coefficient**	**Std. Err.**	**z**	***p*-value**
DDR [L1]	0.0201	0.0187	1.0800	0.2820
DREN [L1]	−0.0744	0.0133	−5.5900	<0.001
GDP [L1]	−0.0002	0.0001	−1.3900	0.1630
CO_2_ [L1]	<0.001	<0.001	−2.5200	0.0120
GHE [L1]	−0.0102	0.0026	−3.8900	<0.001

**Table 11:** Fixed-effects estimates with Driscoll–Kraay standard errors.

Variable	Coefficient	Std. Error	t-Statistic	*p*-Value
L1.DLEB	0.0276	0.0557	0.5000	0.6250
DDR	−0.0899	0.0176	−5.1000	<0.001
DREN	0.0105	0.0079	1.3300	0.1970
GDP	0.0003	0.0001	1.7700	0.0910
CO_2_	<0.001	<0.001	0.0900	0.9280
GHE	0.0025	0.0015	1.6600	0.1120
_cons	−0.0007	0.0016	−0.4800	0.6360

## Data Availability

The data used in this study are publicly available from the World Bank’s World Development Indicators (WDI) database. The datasets analyzed during the current study can be accessed at: https://databank.worldbank.org/source/world-development-indicators (accessed on 26 January 2026).

## Appendix A

**Table A1:** Literature Synthesis on Environmental Quality, Renewable Energy, Healthcare Expenditure, and Life Expectancy.

Authors	Country/Region	Methodology	Main Variables	Key Findings
Preston (1975) [2]	Cross-country	Econometric analysis	Income, mortality, life expectancy	Economic development significantly improves life expectancy, especially in lower-income countries.
Cutler et al. (2006) [1]	Global	Literature review/econometric synthesis	Income, healthcare, mortality	Longevity improvements are strongly linked to healthcare access, education, and economic progress.
Pope et al. (2002) [5]	United States	Cohort analysis	Air pollution, mortality	Long-term exposure to air pollution increases cardiopulmonary mortality and reduces life expectancy.
Markandya et al. (2018) [6]	Global	Policy simulation analysis	Renewable energy, air pollution, health	Renewable energy transition generates significant health co-benefits through pollution reduction.
Polcyn et al. (2023) [19]	Asia	Panel-data analysis	Health expenditure, energy use, pollution, life expectancy	Environmental pollution negatively affects longevity, while healthcare spending improves life expectancy.
Uddin et al. (2023) [17]	Asia	Panel econometric analysis	GDP, healthcare, environmental quality, life expectancy	Environmental degradation and weak healthcare systems reduce life expectancy.
Karimi Alavijeh et al. (2024) [24]	G7 countries	Method of moments quantile regression	Renewable energy, life expectancy	Renewable energy consumption positively influences life expectancy across advanced economies.
Xie et al. (2023) [23]	China	Environmental-health modeling	Renewable energy, air quality, mortality	Renewable energy expansion improves air quality and reduces pollution-related health risks.
Mailloux et al. (2022) [22]	United States	Air quality simulation analysis	PM_2.5_ emissions, health outcomes	Reducing energy-related emissions substantially improves public health outcomes.
Wang et al. (2023) [26]	Cross-country	Panel-data analysis	Renewable energy, environmental quality, life expectancy	Clean energy transition contributes positively to life expectancy through environmental improvements.
Alqadasi et al. (2024) [16]	GCC countries	Public health assessment	Non-communicable diseases, mortality	GCC countries face increasing health burdens associated with NCDs and lifestyle-related diseases.
Islam & Ali (2024) [25]	Saudi Arabia	Policy and sustainability analysis	Green energy transition, sustainability	Successful renewable energy transition requires strong environmental governance and policy coordination.
Abid, Hechmi, & Chaabouni (2024) [15]	GCC countries	Panel-data analysis	Energy intensity, CO_2_ emissions, economic growth	Environmental degradation and energy intensity influence economic and sustainability outcomes in GCC economies.
Nixon & Ulmann (2006) [34]	OECD countries	Panel econometric analysis	Health expenditure, life expectancy	Public healthcare expenditure significantly improves longevity and population health.
Bokhari et al. (2007) [35]	Developing countries	Dynamic panel estimation	Health expenditure, mortality	Increased government health spending improves healthcare outcomes and reduces mortality rates.
